# A comparison of cardiovascular and pulmonary morbidities and risk factors in breast cancer survivors compared to an age-matched female control group in the Lifelines prospective population cohort

**DOI:** 10.1016/j.breast.2023.06.002

**Published:** 2023-06-08

**Authors:** D.S. Spoor, V.A.B. van den Bogaard, N.M. Sijtsema, P. Van der Meer, G.H. de Bock, J.A. Langendijk, J.H. Maduro, A.P.G. Crijns

**Affiliations:** a Department of Radiation Oncology, University of Groningen, University Medical Center Groningen, Groningen, the Netherlands; b Department of Cardiology, University of Groningen, University Medical Center Groningen, Groningen, the Netherlands; c Department of Epidemiology, University of Groningen, University Medical Center Groningen, Groningen, the Netherlands

**Keywords:** Survivorship, Age-matched control group, Late treatment-related toxicity, Modifiable risk factors

## Abstract

**Purpose:**

To provide more insight into late treatment-related toxicities among breast cancer (BC) survivors by comparing morbidities and risk factors between BC survivors and age-matched controls.

**Materials and methods:**

All female participants diagnosed with BC before inclusion in Lifelines, a population-based cohort in the Netherlands, were selected and matched 1:4 to female controls without any oncological history on birth year. Baseline was defined as the age at BC diagnosis. Outcomes were obtained from questionnaires and functional analyses performed at entry to Lifelines (follow-up 1; FU1) and several years later (FU2). Cardiovascular and pulmonary events were defined as morbidities that were absent at baseline but present at FU1 or FU2.

**Results:**

The study consisted of 1,325 BC survivors and 5,300 controls. The median period from baseline (i.e., BC treatment) to FU1 and FU2 was 7 and 10 years, respectively. Among BC survivors more events of heart failure (OR: 1.72 [1.10–2.68]) and less events of hypertension (OR: 0.79 [0.66–0.94]) were observed. At FU2, more electrocardiographic abnormalities were found among BC survivors compared to controls (4.1% vs. 2.7%, respectively; p = 0.027) and Framingham scores for the 10-year risk of coronary heart disease were lower (difference: 0.37%; 95% CI [-0.70 to -0.03%]). At FU2, BC survivors had more frequently a forced vital capacity below the lower limit of normal than controls (5.4% vs. 2.9%, respectively; p = 0.040).

**Conclusion:**

BC survivors are at risk of late treatment-related toxicities despite a more favourable cardiovascular risk profile compared to age-matched female controls.

## Highlights

• Breast cancer (BC) survivors had less hypertension and lower Framingham risk scores compared to age-matched controls.
• BC survivors also had more events of heart failure, conduction disorders and reduced pulmonary capacity.
• Despite a more favourable cardiovascular risk profile, BC survivors are at risk of late treatment-related toxicities.

## 1 Introduction

Breast cancer (BC) is the most common form of cancer in women in the Netherlands with an annual incidence of approximately 15,000 new cases of invasive BC and 2,300 cases of in situ carcinoma [1]. Early detection, particularly through national BC screening, combined with more effective systemic and local therapies, such as radiotherapy (RT), has substantially improved BC survival rates. Consequently, the prevalence of BC survivors, who are at risk of late treatment-related toxicities [2], is increasing.

Radiation-induced cardiac toxicities among BC survivors include a wide spectrum of effects on the heart ranging from pre-clinical findings to symptomatic cardiovascular diseases (CVD), and even mortality [[3], [4], [5]]. Numerous studies have shown that BC survivors have an increased risk of subclinical myocardial dysfunction, electrocardiographic (ECG) abnormalities, acute coronary events, congestive heart failure, valvular heart disease, pericardial disease and cardiac death [[6], [7], [8], [9], [10], [11], [12], [13]]. Previous studies also showed a relationship between chemotherapy for BC treatment (i.e., anthracyclines) and congestive heart failure [2,[14], [15], [16]]. BC treatment-related lung toxicities have been studied less extensively. Nonetheless, despite limited toxicity to the respiratory system with different radiation therapy techniques, acute and long-term pulmonary function changes after BC treatment have been reported [[17], [18], [19], [20], [21]].

Every additional CVD risk factor present at BC diagnosis is associated with an increased risk of cardiac events and death after treatment [22]. Also, the overall survival of cancer survivors who develop CVD is poor and for older BC survivors who survive at least several years, the risk of CVD mortality is even higher than dying from cancer [[23], [24], [25]]. In fact, despite an increased risk of dying from cancer, for older BC patients a less toxic treatment could even benefit the overall survival due to a reduction in CVD mortality [26].

Lifelines is a multi-disciplinary prospective population-based cohort study examining in a unique three-generation design the health and health-related behaviours of 167,729 persons living in the North of the Netherlands which allows for cross-sectional, longitudinal and observational research that can be translated to the general population [27]. It employs a broad range of investigative procedures in assessing the biomedical, socio-demographic, behavioural, physical and psychological factors which contribute to the health and disease of the general population, with a special focus on multi-morbidity and complex genetics [[27], [28], [29]].

The aim of this study was to gain more insight into CVD burden of BC survivors. This was done by comparing cardiovascular and pulmonary morbidities and risk factors (including modifiable lifestyle factors like BMI, smoking behaviour and physical activity) between BC survivors and an age-matched female control group without any oncological history.

## 2 Material and methods

### 2.1 Study population

The overall design of the Lifelines Cohort Study has been described in detail elsewhere [28,29]. In summary, individuals living in the northern part of the Netherlands were invited through their general practitioners (GP) or they could register themselves via the Lifelines website. At inclusion and at follow-up assessments, participants completed a comprehensive set of questionnaires and underwent a series of measurements including physical examination, ECG, laboratory tests and spirometry.

In total, 167,729 participants were included in Lifelines between 2006 and 2013. For this study we selected all female participants who self-reported^2^ a diagnosis of (non-)invasive BC before entry to Lifelines. Participants were excluded when they had a history of other malignancies, excluding nonmelanoma skin cancer, or a second primary tumour. Every BC survivor was randomly matched to four control subjects (i.e., females with an identical age but without an oncological history before entry to Lifelines). This resulted in a population-based cohort of 1,325 BC survivors and 5,300 controls. The period of BC treatment ranged from 1960 to 2014 and 70% was treated after 2000.

### 2.2 Study design

**Fig. 1:**
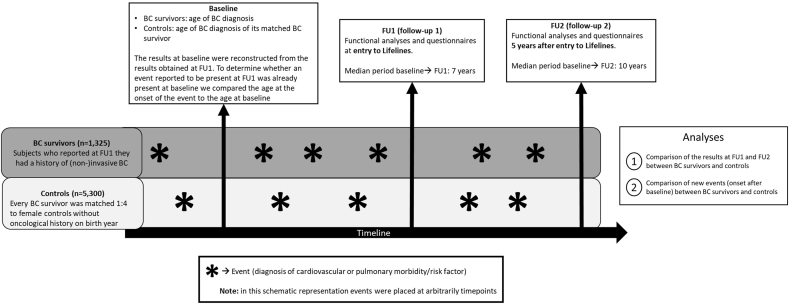
Theoretical example of toxicity events to illustrate the study design. Abbreviations: BC: breast cancer, FU: follow-up.

Fig. 1 visualizes the study design used to compare BC survivors and controls. Functional analyses were performed at entry to Lifelines (FU1) and (for most participants) repeated after five years (FU2).

First, the results of functional analyses at FU1 and FU2 were compared between both groups. Functional analyses at FU2 were voluntary and the period between FU1 and FU2 differed between subjects. To exclude a biased comparison at FU2, the compliance rates, age at FU2 and period between baseline and FU2 were compared for BC survivors and controls.

For BC survivors, baseline was defined as the age of BC diagnosis and for controls as the age of BC diagnosis of its matched BC survivor. To determine whether morbidities, reported at FU1, were already present before baseline we compared the age of onset of this morbidity to the age at baseline. Events were defined as morbidities that were absent at baseline but present at FU1 or FU2. In a second analysis, cardiovascular and pulmonary events were compared between both groups. However, events that occurred between baseline and FU1 and between FU1 and FU2 were only pooled if no bias between both groups at FU2 was identified. Subjects were left-out from the population-at-risk when a morbidity was reported before baseline or when the endpoint was missing.

### 2.3 Data collection

Functional analyses used for this study included anthropometric tests, blood pressure analysis (average of the final three readings in mmHg with Dynamap, PRO 100V2), spirometry (1 measurement with Welch Allyn version 1.6.0.489), ECG (1 measurement with Welch Allyn DT100, 12 leads) and clinical chemistry analyses on blood [29].

The questionnaires that were used in this study provided information on cardiovascular and pulmonary morbidities and risk factors (including BMI, smoking and physical activity). Definitions for cardiovascular (e.g., arrhythmia, atrial fibrillation, myocardial infarction, coronary revascularization and heart failure) and pulmonary morbidities (i.e., asthma or COPD) were based on the ICD-10 guidelines [29]. Endpoints were defined as present when reported in the questionnaire. Results from the functional analyses were then used to validate these self-reported morbidities [30]. For example, self-reported heart failure was validated by the condition that either drug use related to heart failure, pacemaker or ICD implantation or heart transplantation was also reported.

The forced vital capacity (FVC) and forced expiratory volume in 1 s (FEV1) were obtained from spirometry. Test results were compared to subject-specific predicted values, and lower and upper limits of normal (LLN and ULN, respectively) as outcomes depend on age, standing height, sex and ethnicity [31].

Potential ECG abnormalities were automatically identified by an algorithm and then confirmed by a research nurse who consulted a cardiologist to discuss complex cases. ECG abnormalities were categorized by an observer into abnormal rhythm, conduction problems, signs of ischemia, signs of left ventricular hypertrophy or other abnormalities.

Finally, Framingham scores for the 10-year risk of coronary heart disease (CHD) [32] or cardiovascular diseases (CVD) [33] as well as Reynolds Risk Scores [34] were calculated. Use of antihypertensives was not registered at FU2 and therefore Framingham scores for the 10-year CVD risk at FU2 were based on use of antihypertensives at FU1. Also, hsCRP serum analyses at FU1 were used to calculate Reynolds risk scores at FU2 as this was not collected at FU2. Finally, prevalence of metabolic syndrome was evaluated according to the International Diabetes Federation guidelines [35].

### 2.4 Statistical analysis

Differences between BC survivors and controls in terms of baseline characteristics and functional analyses at both FU1 and FU2 were evaluated with two-sided independent samples t-tests or Wilcoxon rank tests for continuous outcomes whenever appropriate, and a Chi-squared test for categorical outcomes. Incidences of cardiovascular or pulmonary events among BC survivors and controls were compared by Odds ratios (OR) and p-values were obtained from Chi-squared tests. For all analyses, p-values < 0.05 were considered statistically significant. Calculations were performed in SPSS software (version 22; SPSS, Chicago, IL).

## 3 Results

Cardiovascular and pulmonary endpoints at baseline were comparable for BC survivors and controls (Table 1). However, at baseline arrhythmia was reported more often among BC survivors (p = 0.022) and therefore this subjective endpoint was left out from further analysis. Instead, potential conduction disorders were evaluated by the objective endpoint ECG abnormalities.

**Table 1:** Cardiovascular and pulmonary endpoints at baseline (i.e., morbidities before BC treatment).

Variables	Female breast cancer survivors (n = 1,325)	Age-matched female controls (n = 5,300)	*P*-value^a^
**General**			
Age, years			
Median	48	48	NA
Range	23–85	23–85	
**Cardiac morbidity, n (%)**			
Arrhythmia^a^			
Yes	109 (8.2)	341 (6.4)	
No	1,212 (91.5)	4,929 (93.0)	**0.022**
Missing	4 (0.3)	30 (0.6)	
Ischemic heart disease^b^, ^c^	**-**	**-**	0.722
Heart failure^b^	**-**	**-**	0.409
**Pulmonary morbidity, n (%)**			
Asthma			
Yes	58 (4.4)	287 (5.4)	
No	1,257 (94.9)	4,949 (93.4)	0.120
Missing	10 (0.8)	64 (1.2)	
COPD			
Yes	39 (2.9)	183 (3.5)	
No	1,259 (95.0)	4,978 (93.9)	0.339
Missing	27 (2.0)	139 (2.6)	
**Cardiovascular risk factors, n (%)**			
Hypertension			
Yes	260 (19.6)	1,028 (19.4)	
No	1,040 (78.5)	4,181 (78.9)	0.830
Missing	25 (1.9)	91 (1.7)	
Diabetes Mellitus			
Yes	25 (1.9)	108 (2.0)	
No	1,298 (98.0)	5,185 (97.8)	0.727
Missing	2 (0.2)	7 (0.1)	
Hypercholesterolemia			
Yes	111 (8.4)	405 (7.6)	
No	1,214 (91.6)	4,851 (91.5)	0.416
Missing	0 (0.0)	44 (0.8)	
Current smoker			
Yes	263 (19.8)	1,067 (20.1)	
No	1,062 (80.2)	4,233 (79.9)	0.818
Missing	0 (0.0)	0 (0.0)	

Abbreviations: COPD: chronic obstructive pulmonary disease, CV: cardiovascular.

a P-value based on two-sided Chi-squared test, significant differences (p < 0.05) are highlighted in bold.

b Exact number not shown to avoid traceability (n < 10 in at least one of the categories).

c Women with a history of ischemic heart disease were defined as those for whom myocardial infarction or coronary revascularization was stated.

Measurements at FU1 and FU2 were performed after a median period from baseline of 7 and 10 years, respectively. Compliance rates at FU2 were higher in the control group compared to the BC survivor group (70.6% vs. 66.5%, respectively; p = 0.003) which could be resulting from a difference in mortality (causes of death were not available) at FU2 (5.1% for BC survivors compared to 1.3% for controls, p < 0.001). However, no differences in the median period between baseline and FU2 (p = 0.231) or median age at FU2 (p = 0.778) were found between both groups and therefore they were considered comparable at FU2.

Results of functional analyses at FU1 and FU2 are presented in Table 2. Compared to controls, BC survivors more frequently used medication prescribed by a doctor more (p < 0.001). They also had higher HbA1c levels at FU1 (p < 0.001) and FU2 (p = 0.002). However, additional analysis showed that based on a cut-off of 42 mmol/mol, in both groups equivalent number of subjects had elevated HbA1c levels (p = 0.145 at FU1; p = 0.427 at FU2). Triglycerides levels were higher for BC survivors at FU1 (p = 0.003) but no differences were found at FU2 (p = 0.923). Except for the diastolic blood pressure at FU2 (p = 0.323), BC survivors had lower blood pressures than the controls. At FU2, BC survivors had more conduction problems (p = 0.021), more frequently a FVC < LLN (p = 0.040) and lower Framingham scores for the 10-year risk of CHD (p = 0.043).

**Table 2:** Results of Lifelines measurements in BC survivors (n = 1,325) and an age-matched female control group (n = 5,300). FU1 and FU2 were performed after a median period after baseline (i.e., BC diagnosis) of 7 and 10 years, respectively.

Variables	Results at FU1			Results at FU2		
	BC survivors	Age-matched female controls	*P*-value	BC survivors	Age-matched female controls	*P*-value
**General**						
BMI (kg/m^2^)						
Mean	26.65	26.62		26.55	26.59	
Std. dev.	4.49	4.53	0.730	4.42	4.59	0.948
Missing, n (%)	1 (0.1)	3 (0.1)		463 (34.9)	1,608 (30.3)	
Physical activity: days per week >30 min						
Mean	4.57	4.51		4.77	4.84	
Std. dev.	2.24	2.22	0.312	2.07	2.00	0.591
Missing, n (%)	148 (11.2)	644 (12.2)		834 (62.9)	3,401 (64.2)	
Active smoker, n (%)						
Yes	170 (13.2)	775 (15.0)		71 (8.1)	342 (9.1)	
No	1,122 (86.8)	4.408 (85.0)	0.102	810 (91.9)	3,401 (90.9)	0.313
Missing	33 (2.5)	117 (2.2)		444 (33.5)	1,557 (29.4)	
Use of medication prescribed by a doctor, n (%)						
Yes	895 (72.4)	2,938 (59.6)		766 (71.7)	2,563 (59.4)	
No	341 (27.6)	1,994 (40.4)	**<0.001**	303 (28.3)	1,751 (40.6)	**<0.001**
Missing	89 (6.7)	368 (6.9)		256 (19.3)	986 (18.6)	
**Laboratory results**						
Total cholesterol (mmol/L)						
Mean	5.53	5.48		5.49	5.43	
Std. dev.	0.98	1.03	0.062	0.97	1.00	0.086
Missing, n (%)	85 (6.4)	101 (1.9)		567 (42.8)	1,794 (33.8)	
HDL cholesterol (mmol/L)						
Mean	1.67	1.68		1.72	1.70	
Std. dev.	0.42	0.42	0.640	0.42	0.44	0.209
Missing, n (%)	85 (6.4)	101 (1.9)		567 (42.8)	1,794 (33.8)	
LDL cholesterol (mmol/L)						
Mean	3.50	3.47		3.55	3.51	
Std. dev.	0.94	0.96	0.302	0.93	0.93	0.260
Missing, n (%)	85 (6.4)	101 (1.9)		567 (42.8)	1,794 (33.8)	
Glucose (mmol/L)						
Mean	5.15	5.13		5.18	5.14	
Std. dev.	1.04	0.98	0.490	1.20	0.98	0.342
Missing, n (%)	92 (6.9)	127 (2.4)		577 (43.5)	1,828 (34.5)	
HbA1C (mmol/mol)						
Mean	39.61	38.97		38.95	38.35	
Std. dev.	5.92	5.52	**<0.001**	5.47	5.06	**0.002**
Missing, n (%)	252 (19.0)	832 (15.7)		571 (43.1)	1,800 (34.0)	
Triglycerides (mmol/L)						
Mean	1.21	1.15		1.18	1.18	
Std. dev.	0.68	0.62	**0.003**	0.62	0.70	0.923
Missing, n (%)	85 (6.4)	101 (1.9)		567 (42.8)	1,794 (33.8)	
**Blood pressure**						
Systolic, mmHg						
Mean	127.5	128.6		131.3	133.5	
Std. dev.	17.1	17.5	**0.034**	17.5	18.4	**0.002**
Missing, n (%)	11 (0.8)	9 (0.2)		472 (35.8)	1,616 (30.5)	
Diastolic, mmHg						
Mean	72.8	73.4		72.9	73.2	
Std. dev.	9.1	9.1	**0.041**	9.0	9.1	0.323
Missing, n (%)	11 (0.8)	9 (0.2)		472 (35.8)	1,616 (30.5)	
**ECG, n (%)**						
Normal	1,286 (97.2)	5,139 (97.1)		825 (95.9)	3,588 (97.3)	
Abnormal	37 (2.8)	152 (2.9)	0.978	36 (4.1)	99 (2.7)	**0.020**
Missing	2 (0.2)	9 (0.2)		464 (35.0)	1,613 (30.4)	
*ECG abnormality*, *n (%)*						
Abnormal rhythm	10 (0.8)	48 (0.9)	0.597	12 (1.4)	36 (1.0)	0.279
Conduction problems	13 (1.0)	47 (0.9)	0.746	13 (1.5)	26 (0.7)	**0.021**
Other	14 (1.1)	57 (1.0)	–	10 (1.1)	34 (1.0)	–
**Spirometry**						
FVC, L						
Mean	3.58	3.59		3.45	3.45	
Std. dev.	0.65	0.64	0.633	0.73	0.66	0.568
Missing, n (%)	455 (34.3)	1,820 (34.3)		1,049 (79.2)	4,132 (78.0)	
FVC < LLN [24], n (%)						
Yes	21 (2.9)	55 (1.9)		15 (5.4)	34 (2.9)	
No	711 (97.1)	2,883 (98.1)	0.090	261 (94.6)	1,123 (97.1)	**0.040**
Missing	593 (44.8)	2,362 (44.6)		1,049 (79.2)	4,143 (78.2)	
FEV1, L						
Mean	2.68	2.68		2.55	2.55	
Std. dev.	0.54	0.54	0.864	0.56	0.53	0.507
Missing, n (%)	455 (34.3)	1,820 (34.3)		1,049 (79.2)	4,132 (78.0)	
FEV1< LLN [24], n (%)						
Yes	52 (7.1)	182 (6.2)		23 (8.3)	96 (8.3)	
No	680 (92.9)	2,756 (93.8)	0.368	253 (91.7)	1,061 (91.7)	0.984
Missing	593 (44.8)	2,362 (44.6)		1,049 (79.2)	4,143 (78.2)	
**Cardiovascular risk score**						
Framingham risk scores						
*10-year CHD risk, %* [25]						
Mean	5.10	5.11		5.77	6.14	
Std. dev.	3.70	3.79	0.999	3.87	4.25	**0.043**
Missing, n (%)	152 (11.5)	377 (7.1)		635 (47.9)	2,104 (39.7)	
*10-year CVD risk, %* [26] a						
Mean	7.36	7.47		9.04	9.55	
Std. dev.	5.56	6.01	0.841	6.38	7.04	0.208
Missing, n (%)	91 (6.9)	117 (2.2)		571 (43.1)	1,808 (34.1)	
Reynolds risk score, % [27] b						
Mean	3.16	3.02		4.56	4.44	
Std. dev.	4.95	4.47	0.243	6.70	5.98	0.707
Missing, n (%)	869 (65.6)	3,411 (64.4)		1,017 (76.8)	3,513 (66.3)	
Metabolic syndrome, n (%) [28]						
Yes	345 (27.5)	1,402 (26.9)		213 (27.6)	1,082 (30.5)	
No	909 (72.5)	3,805 (73.1)	0.675	558 (72.4)	2,462 (69.5)	0.111
Missing	71 (5.4)	93 (1.8)		554 (41.8)	1,756 (33.1)	

Abbreviations: ECG: electrocardiography, FVC: forced vital capacity, LLN: lower limit of normal, FEV1: forced expiratory volume in 1 s, CV: cardiovascular, CHD: coronary heart disease, CVD: cardiovascular disease, CHD: coronary heart disease, CVD: cardiovascular diseases.

Significant differences (p < 0.05) based on a two independent samples *t*-test for continuous outcomes and a Chi-squared test for categorical outcomes between both groups are highlighted in bold.

a Framingham scores for the 10-year CVD risk at FU2 were based on antihypertensives use at FU1.

b The results of hsCRP serum analysis performed at FU1 were used to calculate Reynolds risk scores at FU2.

Table 3 shows the events (i.e., cardiovascular or pulmonary morbidities and risk factors) observed during follow-up (i.e., between baseline and FU1 or, if available, FU2). Compared to controls, more incidences of heart failure (p = 0.017) and less incidences of hypertension (p = 0.009) were found among BC survivors. An additional logistic regression analysis (results not shown) revealed that the relationship between BC and heart failure was independent on hypertension at baseline or the Framingham score for the 10-year risk of CHD.

**Table 3:** Incidences (i.e., reported at FU1 or FU2 but absent at baseline) of cardiovascular and pulmonary morbidities among breast cancer survivors and age-matched female controls.

Endpoint	new cases (% of subjects at risk)		OR (95% CI)	*P*-value^a^
	BC survivors (n = 1,325)	Age-matched female controls (n = 5,300)		
Ischemic heart disease^b^	16 (1.2)	71 (1.4)	0.90 (0.52–1.55)	0.702
Heart failure	28 (2.1)	66 (1.3)	1.72 (1.10–2.68)	**0.017**
Asthma	69 (5.5)	268 (5.4)	1.01 (0.77–1.33)	0.918
COPD	39 (3.1)	165 (3.3)	0.93 (0.65–1.33)	0.669
Hypertension	170 (16.3)	833 (19.9)	0.79 (0.66–0.94)	**0.009**
Diabetes Mellitus	43 (3.3)	182 (3.5)	0.94 (0.67–1.32)	0.728
Hypercholesterolemia	152 (12.5)	694 (14.3)	0.86 (0.71–1.04)	0.108
Stopped smoking	207 (78.7)	806 (75.3)	1.22 (0.88–1.68)	0.241

Abbreviations: FU: follow-up, BC: breast cancer, OR: odds ratio, COPD: chronic obstructive pulmonary disease.

a *P*-value based on two-sided Chi-squared test, significant differences (p < 0.05) are highlighted in bold.

b Women with a history of ischemic heart disease were defined as those for whom myocardial infarction or coronary revascularization was stated.

## 4 Discussion

In this study we evaluated potential late BC treatment-related toxicities for a population-based cohort including 1,325 BC survivors and 5,300 age-matched female controls without oncological history. Overall, only limited differences were found between both groups. However, compared to controls BC survivors had more diagnoses of heart failure, reduced pulmonary function and ECG abnormalities. In contrast, less incidences of hypertension, lower blood pressures and lower Framingham scores for the 10-year risk of CHD were observed among BC survivors.

Unfortunately, treatment information was not available and therefore we were not able to confirm that heart failure, conduction disorders and impaired pulmonary function should be considered late BC treatment-related toxicities. However, these findings are in line with other studies.

For example, chemotherapy for BC treatment (i.e., anthracyclines) has a dose-dependent association with congestive heart failure [14,15]. An increased risk of heart failure was also observed in women treated for BC RT compared to controls without cancer [2]. Different studies found a dose-dependent association between incidental radiation of the heart and subclinical left ventricular dysfunction [36,37]. Therefore, the increased risk of heart failure for BC survivors compared to the controls is probably attributed to previous treatment with chemotherapy and RT.

At 10 years after BC diagnosis, but not at 7 years after BC diagnosis, more ECG abnormalities (mostly conduction disorders) were observed among BC survivors. According to Jaworski et al. [3] conduction abnormalities like atrioventricular block, sick sinus syndrome, prolonged QTc, supraventricular arrhythmias and ventricular tachycardia might manifest years or even decades after exposure to thoracic irradiation. The increased rate of ECG abnormalities at FU2 could be therefore a late effect of incidental heart radiation.

RT for BC can cause radiation pneumonitis and a subsequent decline in FVC and FEV1 during the first months after RT, which is often fully recovered after one year [17]. Erven et al. [18] found that after this recovery another decline in pulmonary function was observed between 8 and 10 years after treatment, which is likely a result of accelerated lung fibrosis. This could explain why we found a FVC below LLN more frequently among BC survivors than controls at FU2 but not at FU1.

Also, endocrine therapy plays a role in the risk of long-term cardiovascular morbidity. Tamoxifen is associated with an increased risk of thromboembolic events blood clots and aromatase inhibitors effect lipid profiles and may inhibitors increase cholesterol levels [38,39].

Our results showed that, compared to the controls, BC survivors had less diagnoses of hypertension within the first 10 years after baseline and lower Framingham risk scores for the 10-year risk of CHD at 10 years after baseline. Kwan et al. [40] found an increased rate of hypertension among BC survivors compared to controls at 2 years after diagnosis but a trend towards less hypertension 10 years after BC diagnosis. The initial risk of hypertension was higher for BC survivors treated with left-sided RT and hormonal therapy, which suggests it is related to incidental radiation of the heart with BC RT, but they did not clarify why the initial risk of hypertension disappeared over time. Besides, up to 10 years after treatment, BC survivors were more frequently diagnosed with diabetes [40]. In line with these findings, changes in insulin resistance after adjuvant BC treatment have been found [41]. In our study HbA1c levels, a measure of the average blood glucose levels over the last 2–3 months, were 0.6 mmol/mol higher for BC survivors (FU1 and FU2) but this was not reflected by an increased incidence of diabetes. At FU2 metabolic syndrome was observed in 27.6% of the BC survivors compared to 30.5% controls. However, the difference was not significant (p = 0.111).

Adapting a healthier lifestyle, for example by increased physical activity, a prudent diet and weight maintenance, reduces the risk of cardiovascular disease [[42], [43], [44]]. Therefore, the lower incidence of hypertension and lower Framingham score for the 10-year risk of CHD among BC survivors might be explained by lifestyle changes after BC diagnosis. However, as shown in Table 3, we did not find any differences between BC survivors and controls with respect to modifiable lifestyle risk factors like BMI, physical activity and smoking behaviour. Studies have shown that BC survivors have different concerns which can be both motivating and deterring with regard to adaptation of a healthier lifestyle [45]. BC survivors experience higher levels of stress and lower mental functioning compared to the general population, but little is known about how this affects exercise behaviour. Only few BC survivors meet recommendations for physical activity or fruit and vegetable consumption [46].

Another explanation for small but favourable difference in cardiovascular risk factors is that BC survivors have better access to preventive care. We found that BC survivors used medication prescribed by a doctor more often than controls but contradicting evidence was found regarding this hypothesis. In the majority of preventive medical care that was provided in the U.S. cancer survivors had more frequent screening compared with subjects without history of cancer [47]. On the other hand, the provision and uptake of preventive care in the U.K. was comparable between cancer survivors and controls without a history of cancer [48]. Finally, it was shown that the amount of care that BC survivors received on acute or late conditions was equivalent to controls [49].

Overall, we only found limited differences between BC survivors and controls, which is in line with previous studies. For example, Boerman et al. [16] did not find an increased risk of CVD resulting from BC treatment with RT or chemotherapy. However, several studies, like the study by Greenlee et al. [2], identified cardiovascular morbidities related to the BC treatment. Gernaat et al. [50] found that among women with low Framingham risk scores, patients receiving BC treatment were at higher risk of hospitalization or death from a CVD event compared to subjects who did not receive cancer treatment. Boekel et al. [51] showed that RT may worsen acute coronary syndrome prognosis. Finally, Yandrapalli et al. [52] found that at the moment of a first myocardial infarction, patients with a history of BC were older and had less cardiovascular risk factors compared to patients without a history of BC.

In this study is we evaluated a wide variety of cardiovascular and pulmonary endpoints in a population-based cohort of BC survivors and age-matched controls. Self-reported endpoints, obtained from questionnaires, were validated by the results of functional analyses. However, the study also has some limitations. First, reconstruction of baseline outcomes was only possible for binary endpoints with a specified date of diagnosis. Therefore it was not possible to reconstruct baseline values for continuous outcomes like laboratory results. Also, the results at FU2 were likely affected by mortality as the mortality rate at FU2 was approximately four times higher for BC survivors than controls. Although cause of death was not reported, this difference is likely a result of BC- and CVD-related mortality. For example, BC progression was reported at FU1 by 60% of the BC survivors who were deceased at FU2. Other studies found that after more than seven years following treatment the risk of CVD-related death is approximately 80% higher for BC survivors than women without BC [53]. Consequently, the additional risk of severe CVD morbidity among BC survivors may be higher than captured in this study [53]. However, a sub-analysis on BC survivors who reported at FU1 to be cured from BC and their matched controls showed similar results (i.e., more incidences of heart failure and less incidences of hypertension were found among BC survivors). Finally, as information on the BC treatment was not available it was not possible to evaluate associations between BC treatment factors and potential treatment-related toxicities. Further exploration of such associations requires regression modelling in large cohort studies, such as the MEDIRAD BRACE project [54].

## 5 Conclusion

Up to 10 years after BC treatment heart failure, conduction disorders and impaired pulmonary capacity were more common among BC survivors compared to age-matched female controls without oncological history. On the other hand, BC survivors also had less incidences of hypertension and lower Framingham scores for the 10-year risk of CHD.

## Funding

The Lifelines initiative has been made possible by subsidy from the Dutch Ministry of Health, Welfare and Sport, the Dutch Ministry of Economic Affairs, the University Medical Center Groningen (UMCG), Groningen University and the Provinces in the North of the Netherlands (Drenthe, Friesland, Groningen). Data may be obtained from a third party and are not publicly available. Researchers can apply to use the Lifelines data used in this study. More information about how to request Lifelines data and the conditions of use can be found on their website (https://www.lifelines.nl/researcher/how-to-apply).

## Declaration of competing interest

The department of Radiation Oncology of the University Medical Center Groningen has research collaborations with IBA, Philips and Mirada as well as R&D collaboration agreements with RaySearch, Siemens, Elekta en Leonie. Moreover, author J.L. reports personal fees from 10.13039/100014252IBA.

## Footnotes

2 This research has been performed at the University Medical Center Groningen, the Netherlands.
